# Parents’ experiences of care following the loss of a baby at the margins between miscarriage, stillbirth and neonatal death: a UK qualitative study

**DOI:** 10.1111/1471-0528.16113

**Published:** 2020-02-21

**Authors:** LK Smith, J Dickens, R Bender Atik, C Bevan, J Fisher, L Hinton

**Affiliations:** ^1^ Department of Health Sciences University of Leicester Leicester UK; ^2^ Bereavement Specialist Midwife University Hospitals of Leicester NHS Trust University of Leicester Leicester UK; ^3^ Miscarriage Association Wakefield UK; ^4^ Sands, the Stillbirth and Neonatal Death Charity London UK; ^5^ Antenatal Results and Choices London UK; ^6^ Applied Research, Health Experiences Research Group Nuffield Department of Primary Care Health Sciences University of Oxford Oxford UK

**Keywords:** Bereavement care, miscarriage, neonatal death, qualitative, stillbirth

## Abstract

**Objective:**

To explore the healthcare experiences of parents whose baby died either before, during or shortly after birth between 20^+0^ and 23^+6^ weeks of gestation in order to identify practical ways to improve healthcare provision.

**Design:**

Qualitative interview study.

**Setting:**

England through two parent support organisations and four NHS Trusts.

**Sample:**

A purposive sample of parents.

**Methods:**

Thematic analysis of semi‐structured in‐depth narrative interviews.

**Main outcome measures:**

Parents’ healthcare experiences.

**Results:**

The key overarching theme to emerge from interviews with 38 parents was the importance of the terminology used to refer to the death of their baby. Parents who were told they were ‘losing a baby’ rather than ‘having a miscarriage’ were more prepared for the realities of labour, the birth experience and for making decisions around seeing and holding their baby. Appropriate terminology validated their loss, and impacted on parents’ health and wellbeing immediately following bereavement and in the longer term.

**Conclusion:**

For parents experiencing the death of their baby at the margins between miscarriage, stillbirth and neonatal death, ensuring the use of appropriate terminology that reflects parents’ preferences is vital. This helps to validate their loss and prepare them for the experiences of labour and birth. Reflecting parents’ language preferences combined with compassionate bereavement care is likely to have a positive impact on parents’ experiences and improve longer‐term outcomes.

**Tweetable abstract:**

Describing baby loss shortly before 24 weeks of gestation as a ‘miscarriage’ does not prepare parents for labour and birth, seeing their baby and making memories.

## Tweetable abstract

Describing baby loss shortly before 24 weeks of gestation as a ‘miscarriage’ does not prepare parents for labour and birth, seeing their baby, and making memories.

## Introduction

Miscarriage, stillbirth and neonatal death are intensely painful and traumatic for many parents^1^ and associated with substantial direct and indirect, psychological and social costs to women, families and society.^2^ Wide variability in bereavement care provision^3^, ^4^ prompted the development of the National Bereavement Care Pathway (NBCP; https://nbcpathway.org.uk/). Launched in 2017, it outlines best practice bereavement care following miscarriage (encompassing loss after ectopic and molar pregnancy), stillbirth, termination of pregnancy for fetal anomaly, neonatal death and sudden unexpected death of an infant.

In UK clinical practice, based on birth and death registration law (Box 1), a baby born showing no signs of life is referred to as a ‘miscarriage’ before 24^+0^ weeks and a ‘stillbirth’ from 24^+0^ weeks onwards. ‘Early neonatal death’ refers to a live‐born baby who dies shortly after birth. These categorisations based on gestational age and signs of life, may not align with the realities of parental experience. Here we explore the healthcare experiences of parents whose baby died just before 24 weeks of gestation.

Box 1 **Definitions of UK birth and death registration in the UK by gestational age and live‐birth status**

**Babies born showing signs of life**

***All gestations***

If a baby is born alive regardless of gestation, it is a UK legal requirement that the birth must be certified and subsequently registered.

It is a UK requirement to certify and register deaths of all babies born alive, regardless of the gestation at which the birth occurred. An *early neonatal death* is defined as a death occurring in the first 7 days of life.

**Babies born showing no signs of life**

***Before 24^+0^ weeks of gestation***

If a baby is born showing no signs of life before 24^+0^ weeks of gestation there is no legal certification or registration of the birth or death in the UK. These losses are often referred to as a *miscarriage* or where there has been a decision to end the pregnancy because of fetal health indications, as a *termination of pregnancy for fetal anomaly*. Some losses at this gestation may also be referred to as an *ectopic or molar pregnancy*.

***Births at or after 24^+0^ weeks of gestation***

In the UK if a baby is born showing no signs of life at or after 24^+0^ weeks of gestation there is a legal requirement to register the death as a *stillbirth*.

## Methods

### Sample and recruitment

We undertook semi‐structured narrative interviews with parents whose baby died before, during or shortly after birth at 20^+0^ to 23^+6^ weeks of gestation. An advisory panel of parents, clinicians and parent advocacy groups was involved throughout to ensure appropriateness of study materials and validity of findings. We included experiences of loss following spontaneous antepartum death, termination of pregnancy for fetal anomaly, and preterm birth. We recruited parents retrospectively and prospectively through parent support organisations (Sands and the Miscarriage Association) and clinicians at four participating clinical sites. Information about the study was given to potentially eligible parents, either in person or, in the case of parent support organisations, via social media. Parents who expressed an interest in participating were given further detailed information and contacted by telephone to provide an opportunity for questions. For those parents participating, an interview was arranged in a location of their choosing.

We aimed for a maximum variation purposive sample to ensure that we spoke to parents with a wide range of experiences of loss and demographic characteristics. Interviews took place across England between 1 September 2016 and 30 August 2017. Where both parents participated they were given the option of being interviewed together or individually. The interview started with an open‐ended question asking parents to relate their experiences of loss followed by additional questions from a flexible topic guide developed with parents, researchers and clinicians (see Supplementary material, Appendix S1). Parents were interviewed by LS and/or LH and offered audio and/or video recording.

Interviews were transcribed verbatim and checked. Parents were given the opportunity to review the checked transcript and to remove sections from the analysis. Transcripts were anonymised and coded in nvivov.9 by LS using a coding framework developed with LH. LS undertook the analysis with regular peer debriefing and reflexive conversations with LH. Emerging themes were identified using a ‘modified grounded theory’ approach, and were verified by each researcher and the advisory panel. Summaries of key themes and extracts from interviews were published on the Healthtalk website (www.healthtalk.org/20-24). Participants gave informed written consent before interview and again before analysis and publication. Pseudonyms were assigned to parents when requested.

This article presents independent research funded by the National Institute for Health Research.

## Results

Interviews were undertaken with 38 parents: 10 parent pairs and 18 mothers who had experienced their baby’s death between 20^+0^ and 23^+6^ weeks of gestation (see Supplementary material, Appendix S2). One further parent was interviewed but lost to follow up. Loss had occurred between 6 weeks and 25 years before the interview (see Supplementary material, Appendix S1 and http://www.healthtalk.org/peoples-experiences/pregnancy-children/losing-baby-20-24-weeks-pregnancy/peoples-profiles). Interviews lasted between 39 minutes and 2 hours 45 minutes. This paper focuses on themes around the provision of health care of particular relevance to parents’ experiences following loss at the limit between definitions of ‘stillbirth’, ‘miscarriage’ and ‘neonatal death’. The key themes were identified by the researchers and validated by the patient and public advisory board. These included parents’ preparedness for the birth experience, for seeing their baby and making memories, longer‐term physical and emotional wellbeing and how they might validate their loss in the absence of legal documentation. These themes were strongly mediated by an overarching theme around the terminology used to describe their loss by health professionals and how it impacted on their experiences (detailed parents’ narratives are available in the Supplementary material, Appendix S3).

### The importance of terminology

The parents we spoke to felt strongly that describing their loss as a ‘miscarriage’ was inappropriate and did not adequately describe their lived experience. They appreciated being treated as if they were ‘having a baby’ or ‘losing a baby’. The use of inappropriate terminology impacted on Camille profoundly. She was told she was having a miscarriage as she went into preterm labour at 21 weeks. Her baby showed signs of life for nearly an hour after birth and ‘being told “you're having a miscarriage”… it doesn't prepare you for it’. Sam, whose pregnancy was induced following diagnosis of a fetal anomaly, felt that healthcare professionals treated her son ‘very much like he wasn't a baby, to them, he was just a thing and as a parent, that’s really difficult to hear.’ Parents did not seek to create a hierarchy by suggesting their loss was different to a miscarriage, nor to demean others’ experiences, but felt it important to convey that their personal experience did not align with the word ‘miscarriage’ when experiencing the death of their baby. Carly explained: ‘I'm not trying to demean people who go through miscarriages, or make their grief insignificant. I just think the grief might feel the same, but the experience is so different, and the trauma is a lot different’.

### Being prepared for labour and birth

Many women we spoke to ‘hadn't realised properly that I'd have to actually give birth’ (Alison, pregnancy induced for fetal anomaly). The terminology health professionals used about the baby was extremely important in women’s expectations for labour and birth. The term ‘miscarriage’ added to some women’s distress because they did not associate it with ‘giving birth’ and the pain and physicality of labour. Courtney was unprepared for her experience: ‘so in my head I was like “it's not going to be like this, it's going to be like, like just blood or whatever”. But you know, I had to full on give birth.’

Many women had not been pregnant previously, had not yet attended antenatal classes and so were extremely unprepared for the labour and birth experience. Parents appreciated midwives and other health professionals who helped them understand what labour and birth might be like, how their baby might look and feel, and validated the birth experience. Emily found the birth extremely hard and was reassured when her midwife compared her experience as similar to birth later in pregnancy as ‘that's the majority of the hard work, it's just the same, and you did really well.’

Parents appreciated staff who understood their needs and cared for them with compassion from diagnosis through to labour and birth and beyond. Camille contrasted how she felt she was treated in an early pregnancy unit within a gynaecology department as a ‘woman with a pile of tissue in her uterus’ compared with the maternity unit where she was admitted at 20 weeks, where she felt cared for liked a pregnant woman with a baby.

### Being prepared for seeing the baby

Parents who were treated as if they were having a baby were more prepared for how their baby might look and found it easier to make decisions about seeing and holding their baby. In contrast, the use of the word ‘miscarriage’ by the staff impacted on Camille and her husband’s expectations when seeing their baby as ‘My husband was actually really surprised when they put her in my arms, and he said, “She's a real baby. She's even got hair.”… I don't think either of us were expecting to have a baby. Because we had been told the word “miscarriage” so many times’. Similarly Mike was unprepared for how his daughter looked: ‘it wasn't like a fetus… she was perfectly formed… – that wasn’t a miscarriage, in no way, shape or form’.

### Memory‐making

Memory‐making was often extremely important to parents to help frame their baby’s death and validate their life. Memory‐making included spending time with their baby, and collecting artefacts to remember them by.

Official certificates, or lack of them, were a potent part of this memory‐making process. Parents whose baby was born showing no signs of life before 24 weeks of gestation did not receive an official birth or death certificate. Parents were sometimes offered informal birth and death certificates. Although appreciated by some, for others they were a stark reminder that there was no legal documentation of their baby’s life. Carly felt: ‘It's just like insult to injury… it's just a printed out bit of paper that the hospital gives you, that's not formal. It's not recognised, and it's not official, like a birth and death certificate.*’* These official documents would have validated their baby’s birth and death and allowed the parents access to benefits such as parental leave and pay that are not available to those whose baby is born showing no signs of life before 24^+0^ weeks. This was particularly pertinent to Kirsty: ‘I think out of the whole experience, my hang‐up has been the birth certificate. She was 2 days short. They had a crash team there. We had a baby, but she'll never be recognised by UK law… she just didn't exist.’ None of the parents we spoke to who were required to officially register their live‐born baby’s birth and death discussed any negative impact of this process but described how they valued the access to parental leave and maternity pay. Creating memory boxes with artefacts to remember their baby by was important and helpful to many parents. Items included photographs of their baby, a lock of hair, footprints or handprints, the baby’s wristband, or the blanket their baby was wrapped in. The lack of official documents made items like hospital wristbands extremely important, as they were symbols of an official recognition of their existence. ‘That's like the only real medical documented proof that I have that she was here’ (Carly).

Photographs played an important role. Midwives used them to help prepare some parents for how their baby looked before seeing them face‐to‐face. Sam and Sarah appreciated guidance and gentle encouragement from midwives who supported them after the birth, particularly sharing photos or reassuring them about how their baby looked, ‘because we didn't know what to expect and it's easier to forget a photo than it is something you've actually lived’ (Sam). Parents also kept photographs as a memento, to validate their loss, and used them to help friends and family understand what they had been through. Some parents were unsure whether they wanted to take mementos of their baby home. They appreciated them being stored with their hospital records or given to them in a sealed envelope so they could look at them in the future. David really appreciated encouragement from their midwife: ‘“Do you want me to take pictures? You will, you will appreciate these pictures. Not now, not tomorrow, but in the future.” And they're right.’

Opportunities to make memories were often shortlived because the babies’ bodies deteriorate very quickly at these early gestations. Midwives who facilitated parents spending time with their baby helped to create important memories for the long term. Some parents like Courtney were not supported in memory‐making. They found it hard when they realised the breadth of bereavement care elsewhere through talking to others online or in support groups and what they had missed.

### Postnatal experiences

Losing a baby at this gestation meant women often experienced postnatal physical symptoms, such as bleeding, their breast milk coming in and hormonal swings. Many were unprepared for these physical legacies of birth. Not having a baby to focus on made some even more aware of these symptoms. Many women were physically exhausted and felt ‘bereft’ and ‘panicky’. Several mothers experienced heavy lochia following the birth, which they had not expected. They wished they had been made aware that heavy bleeding might happen and what was a normal amount. Breast milk coming in was an extremely painful reminder of the loss of their baby. Maxine described: ‘your milk comes in like a new mum… your body is so, so cruel, because my body thinks it's just had a baby. And it doesn't have a baby’. Women appreciated when staff discussed options to manage lactation.

### Longer‐term emotional experiences

The terminology used to describe loss also had a longer‐term emotional effect on parents. Being told they were having a baby as opposed to a miscarriage helped parents process their grief after their loss. As already highlighted, many parents found out through support groups that there was a wide variation in healthcare experiences following loss. Sarah felt that she was treated as if she had had a stillborn baby: ‘I knew a lot of other people who'd had babies similar time as us, similar week, but had been treated – you know – not badly, but they felt that they had been treated as if they'd had a miscarriage’ and this made a ‘massive difference to how we dealt with it afterwards’.

Parents sometimes found that when their loss was labelled a ‘miscarriage’ it was harder for them to talk about the loss of their baby with friends and family in the longer term as ‘because she hadn't reached 24 weeks, it wasn't legitimate’(Matthew); this increased their distress and impacted the type of support others offered them. Photographs of the baby were a way of helping friends and family to better understand parents’ experiences as ‘they're like “Oh, my goodness – she is a, like just a tiny little baby.”’(Mike).

## Discussion

### Main findings

This qualitative interview study focussed on parents’ lived experience of the death of their baby a few weeks, days or even hours before the legal definition of stillbirth of 24^+0^ weeks of gestation. The major emergent theme was the impact of the terminology used by healthcare professionals and significant others. Describing loss at this gestation as a ‘miscarriage’ was unhelpful to parents because the physical and emotional reality of losing a baby was very different to their perceptions of miscarriage. In contrast, where health professionals used language such as ‘losing a baby’, parents were more prepared for the realities of the birth experience, both physically and emotionally, and it had a positive impact on their longer‐term experiences of grief. The views of parents and parent advocacy groups were key in ensuring the validity of these themes.

### Strengths and limitations

While there have been studies of parents’ experiences of miscarriage, stillbirth and neonatal death, there has been little focus on loss at the gestational threshold for legal registration of loss. Our research involved parents with a wide range of experiences, outcomes and demographic characteristics, including bereaved parents recruited through support organisations and hospitals. However, we recognise that participants may have been more likely to choose the terms ‘parent’ and ‘baby’ to describe their experience whereas other eligible participants, whose voices we did not hear, may not consider themselves as parents and may refer to their experience as a pregnancy loss or miscarriage, and to their baby as a fetus or pregnancy. It is extremely important that healthcare professionals caring for people in this situation guard against making assumptions, listen carefully and remain sensitive to the language preferred by the woman and her partner with regard to the loss they have experienced. The strong involvement of parents, clinicians and parent advocacy groups in the design and interpretation of findings was extremely important to the validity of the study findings.

### Interpretations

The impact and importance of terminology following pregnancy loss is not new. Historically, spontaneous pregnancy loss was referred to clinically as an ‘abortion’ with negative connotations for women experiencing the loss of a wanted pregnancy.^5^ Influenced by women’s support groups and feminist commentators, the term ‘miscarriage’ has become commonly used.^6^ But at this gestation, the distinction between the terms ‘miscarriage’ and ‘stillbirth’ is not trivial. The term miscarriage focuses attention on the woman’s body failing and denying fetal personhood. Some health professionals may use the term ‘miscarriage’ precisely for these reasons, to, in good faith, minimise the impact of the loss on parents. However, this may have unintended consequences, lessening the importance of parents’ grief, denying the importance of the baby as a loved child and part of the family, and impacting on expectations of birth. Our findings reflect Jonas‐Simpson and McMahon’s finding that the language of loss has the ability to ‘intensify suffering or enhance the family’s experience of grieving’.^7^ They identified positive experiences associated with language that acknowledged the loss of a baby and harmful effects of language that aimed to diminish the loss to ease suffering when the meaning of that loss is significant to parents.

Our findings suggest that the language used impacted on women’s expectations and experiences of labour and birth. Preparing women for labour and birth is a central aspect of midwifery care. Recent UK National Institute for Health and Care Excellence (NICE) guidelines for intrapartum care highlight the importance ‘tone and demeanour, and of the actual words used’ when developing rapport with women during labour.^8^ Sensitive language is key to a positive birth experience for women giving birth (blogs.bmj.com/bmj/2018/02/08/humanising‐birth‐does‐the‐language‐we‐use‐matter; accessed 28 June 2019), and may increase women’s ability to cope and to ‘avoid feelings of loneliness and fear’,^9^ which is vital at this difficult time and may have a long‐term impact on psychological health if misused.

Cassaday^10^ reported how the impact of pregnancy loss on psychological functioning and grief is heightened if it is sudden and unexpected. Parents suffer from the paucity of experiences and memories to share and ‘no concrete object to mourn’. This is pertinent to the parents studied here where there is a lack of legal registration documents and limited time with their baby to make memories, inhibiting validation of their loss. Post‐mortem photographs helped to provide a record of the physical existence of their baby and to establish and share the identity of the baby and facilitate parenthood.^11^

Heazell et al.’s systematic literature review of the economic and psychosocial impact of stillbirth found that many parents experienced ‘longer‐term disenfranchised grief’ following stillbirth, as parents felt their grief was not legitimate or recognised by health professionals, family, friends or wider society. Our interviews reinforce this finding but also suggest that excluding parents from the terminology of stillbirth further intensifies their feelings of marginalisation and grief. Parents we spoke to often felt that they did not know where they fitted in with other parents who had experienced miscarriage and stillbirth. This led to feelings of isolation and, in some cases, disconnection from support groups that offer significant support following loss,^2^ because they felt that they focused on either miscarriage or stillbirth. These feelings of isolation and disconnection from wider support have resonance with broader writings on sociology and health inequalities.^12^ Social roles, defined as ‘behavioural enacting of the patterned expectations attributed to position’, are a core concept of medical sociology. Merton introduced the notion of ‘reference groups’ that dictated norms and values, and social comparison with those of a similar social status. Although his focus was on the professional socialisation of clinicians, rather than on parents caught at the margins of pregnancy loss, his broad analysis on social status and its links to inequalities are pertinent. Merton identified conflict and tensions that arise from unfavourable social comparison and relative deprivation. Parents in our study felt caught at the margins of miscarriage, stillbirth and neonatal death, which impacted on how they asserted a claim to ‘candidacy’ in accessing health services.^13^ Referring to their experience as ‘miscarriage’ impacted on their feelings of eligibility for bereavement support and the wider social understandings of the depth of the experience that they had been through. They felt assigned a social role that did not match up to their experiences, rendering them vulnerable, isolated and unsupported in hospital and wider society.

## Conclusions

With an increase in live births at 22^+0^ to 23^+6^ weeks of gestation receiving neonatal care and surviving to be taken home by their parents^14^, ^15^ and also increases in babies registered as ‘live born’ before 22 weeks of gestation in the UK in recent years (https://www.ons.gov.uk/peoplepopulationandcommunity/birthsdeathsandmarriages/livebirths/datasets/birthcharacteristicsinenglandandwales; accessed 1 July 2019) use of the word ‘miscarriage’ to describe the death of any baby before 24 weeks of gestation in the UK is problematic. In most high‐income countries, the registration criterion for stillbirth is 22^+0^ weeks of gestation or even earlier.^16^ Our findings reinforce the need to use language in healthcare encounters that validates the loss of a baby, acknowledges the hopes and dreams associated with that loss, and prepares parents for the experience of labour and birth (Box 2). However, ‘it will take more than words to truly improve patients’ experiences’^6^ and the use of the ‘correct terminology’ may make some health professionals complacent in their provision of care.^17^ Appropriate language needs to be combined with an empathic approach, ‘seeing through the eyes of those affected’^2^ with staff understanding the experiences and needs of different parents and families to have a positive impact on parents’ experiences and improve longer‐term outcomes in the future.

Box 2 **Recommendations for caring for parents experiencing loss between 20^+0^ and 24^+6^ weeks of pregnancy**

The National Bereavement Care Pathway offers core bereavement care principles to ensure high‐quality care for parents following loss (https://nbcpathway.org.uk/). They highlight that ‘some parents may see late miscarriage and premature labour as being very similar even if some staff may view these as very different situations.’ Additional recommendations are listed here when caring for parents experiencing loss between 20^+0^ and 24^+6^ weeks of pregnancy. As a guiding principle, bereavement care should be equitable and parent‐centred regardless of the gestational age of the baby and whether the baby died before, during or shortly after birth.

**Preferred language**

- Actively listen and take the lead from the woman and her partner regarding preferred language.
- Many people choose to describe themselves as ‘parents’ who are ‘having a baby’ or ‘losing a baby’ in the context of a wanted pregnancy with terms such as miscarriage being potentially distressing. However each situation is unique and others would prefer to be addressed as people rather than parents, for the birth to be referred to as the end of the pregnancy or miscarriage, and for the baby to be described as a fetus.

**Preparation for labour and birth**

- Prepare women as early as possible for the physical and emotional realities of the experience of labour and giving birth, particularly as many will not yet have attended antenatal classes.
- Ensure women have information about different types of pain relief available to them and monitor that pain relief is adequate for their needs.
- Many women find terms such as miscarriage or mini‐birth are not useful in preparing them for the experience of birth.
- If there is a fetal heartbeat the parents should be prepared for the possibility of their baby being born alive and any care that would be provided in these circumstances.

**Seeing and holding the baby and making memories**

- Reassure parents that deciding whether to see and hold their baby is an individual choice with no right or wrong decisions. Parents may appreciate guidance to help make these decisions.
- Prepare parents, before birth if possible, for how their baby may look when they are born. Parents appreciate reassurance about their baby’s appearance and photographs can be a useful way to help parents ready themselves to see and hold their baby.
- Refer to the National Bereavement Care Pathway for recommendations on how to support opportunities for memory making with parents.
- The condition of the baby should be considered when discussing memory‐making opportunities. Early opportunities for discussing photographs are important as the baby’s body may deteriorate quickly.
- If parents are unsure whether they want to take mementos of their baby home, they may appreciate being asked if they would like to consent to them being stored with their hospital records or given to them in a sealed envelope to store at home so that they can look at them if they decide to in the future.
- Allow those parents who wish to spend time with their baby to have as much quiet time as they would like and where possible facilitate subsequent visits for parents to visit their baby after death but prepare them that their baby’s body may deteriorate quickly.

**Follow‐up care**

- Ensure women are aware that they will pass lochia after birth and are aware of what is normal and abnormal and when to seek medical advice.
- Inform women about options for the management of lactation, including non‐pharmacological and pharmacological suppression where requested or the option of continued lactation either for personal or donation choices.
- Provide or signpost parents to appropriate counselling and support following their bereavement.

### Contribution to authorship

LS was responsible for the conception of the study, obtaining funding, planning, delivery, qualitative interviews, analysis of the study and wrote the first draft of the paper. LH contributed to the planning of the study, undertook interviews, qualitative analysis, planning and revision of the first draft of the paper. RBA, CB, JF and JD contributed to the planning of the study, interpretation of results and provided critical feedback on the draft paper. All authors read and approved the final manuscript. LS is the guarantor.

### Disclosure of interests

None declared. Completed disclosure of interests forms are available to view online as supporting information.

### Details of ethics approval

The study was approved by the Berkshire Research Ethics Committee (12/SC/0495 12/09/2012).

### Funding

LKS is funded by a National Institute for Health Research Career Development Fellowship. This article presents independent research funded by the National Institute for Health Research (NIHR). The views expressed are those of the author(s) and not necessarily those of the NHS, the NIHR or the Department of Health and Social Care. LH is supported by the NIHR Biomedical Research Centre, Oxford; grant BRC‐1215‐20008 to the Oxford University Hospitals NHS Foundation Trust and the University of Oxford.

### Acknowledgements

We would like to thank all of the parents that participated in the study (www.healthtalk.org.uk/20-24) and acknowledge the help of clinicians at the four participating Trusts and the support organisations: Miscarriage Association, Sands, Antenatal Results and Choices. We would also like to thank all those people who gave us advice, help and guidance through the project (https://healthtalk.org/losing-baby-20-24-weeks-pregnancy/credits). A special thanks for help from Sophie Almond and Julia Clark at the University of Leicester and Ruth Sanders from Nuffield Department of Primary Care Health Sciences at Oxford University.

## Supporting information


**Appendix S1** **.** Topic guide for parent interviews.

**Appendix S2** **.** Biographical profiles of parents participating in the study.

**Appendix S3** **.** Parents’ narratives of healthcare experiences.
