# Supplementary material for: Parents’ experiences of care following the loss of a baby at the margins between miscarriage, stillbirth and neonatal death: a UK qualitative study
Source: BJOG. 2020 Feb 21;127(7):868–74. doi: 10.1111/1471-0528.16113 (PMC7383869; doi:10.1111/1471-0528.16113)
Supplement: Supplementary file 1 — Appendix S1 . Topic guide for parent interviews. [file BJO-127-868-s001.pdf]

Appendix S1. Topic guide for parent interviews

Introduction
I would like to start by asking you to tell me your story about your pregnancy and the birth and loss of your baby.
I'll then talk to you about some of the things you've raised in more detail.
Finding out about the pregnancy
Was this your first experience of being pregnant? – Tell me about your other experiences
I'd like you to think back to when you found out you were pregnant this time, how did you feel about being pregnant? How did this pregnancy feel compared to your previous pregnancies?
Care in early pregnancy
I'd like you to tell me about the care you received during your pregnancy from when you first found out you were pregnant. Can you tell me about who you saw? Did you start by seeing your GP and then when were you referred to the hospital?
Were there any reasons for you to be treated as a high-risk pregnancy at all?
[Dating scan undertaken? Any other interaction with HP at hospital]
How did you feel in yourself at the beginning, can you describe any health problems you had during the pregnancy? I'd like you to now tell me about the appointments you had at the hospital and any scans you received.
Often early in pregnancy you might think about and plan how you'd like the birth to be, can you describe any plans you'd made for the birth of your baby.
Was there anything at this time that made you think you might give birth early in pregnancy?
What did you know about premature birth before you were pregnant, had any other family members or friends had their baby prematurely?
First awareness of possible preterm birth
Can you describe what events or feelings made you start to think that you might give birth early in your pregnancy? Tell me about the time you spent in hospital or being under review
[how many weeks pregnant were they at this stage?]
At that time what kind of discussions did you have with doctors or midwives about the risks of giving birth early in pregnancy?
I'd like you now to think about chats you had with midwife or doctors about the risks of giving birth at this time in your pregnancy and any plans you made together. [did you have any of those conversations]
I'd like you now to think about chats you had with midwives or doctors about the risks of giving birth early in your pregnancy and any plans you made together.
Onset of labour
I'd like you to tell me about when you first thought you might be going into labour or would give birth.
Can you describe to me when and how your labour actually started? Can you talk me through your labour and how it progressed. What health professionals were involved at this stage?
Once you were in labour, can you tell me about when a midwife or doctor talked to you about the possible risks of giving birth this early in pregnancy?
I'd like you to describe to me who came to talk to you about the risks of giving birth early? [midwives, neonatologists, obstetricians] What kind of plan did you discuss for what might happen after the birth? Can you describe what you felt about what you'd been told?
I'd like you to tell me about whether you feel that you agreed a plan with the health professionals together? How did your partner feel about what about the plans that were made?
And at that time how did you feel in terms of confidence about being the one(s) to make a decision about what to do when your baby was born?
The birth of your baby
If it's ok I'd like you now to think about the actual birth of your baby, can you tell me what happened
[Mode of delivery etc]

Can you tell me about the sorts of pain relief you were offered and that you decided to use?
Thinking back to when you were giving birth, tell me about who was looking after you?
Can you describe who else was at the birth of your baby? [Partner, health professionals]
Tell me about what you remember happening after you gave birth to your baby? How did you feel at this time? Can you take me through what happened after your baby was born
Now I'd like you to tell me about whether they tried to resuscitate them. [Where relevant]
How were you feeling while all this was happening?
Neonatal admission – where appropriate
After your baby was born can you tell me about whether they were admitted to a neonatal unit?
How long were they in the NICU? Can you tell me about the care they received there?
Can you take me through all of the decisions you had to make for their care
Tell me about the events that led up to the time of your baby's death
How did the team explain the decisions they made following the birth of your baby?
Can you describe the conversations you had with the team about comfort care for your baby?
The death of your baby
Can you tell me about the moment when your baby died?
Can you tell me who explained to you what had happened to you and your baby and what they told you?
Tell me about how much time you got to spend with your baby. Can you tell me about whether you held your baby after they were born. What events really stood out to you?
Please tell me about any complications you had following the birth?
What did you find helpful in what the doctors told you in those early moments about what had happened to your baby. Can you tell me about what happened next?
How long did you spend in hospital. Can you tell me about the kind of support you received at this time. Where did your baby go after they died?
What plans were discussed about a post mortem? Can you tell me about any plans you made at this stage for a funeral.
How did you feel about leaving the hospital to go home?
Mementoes and leave from work
Often when someone's baby dies, the parents are offered a box of mementoes. Can you describe the memento box if you were given one and whether you found it helpful?
What sort of mementoes have you found helpful?
Not all parents get an official birth certificate and death certificate, what was your experience?
Can you take me through the process you had to go through to register the birth and death of your baby?
Can you tell me about whether you were offered any informal birth and death certificates after the birth?
How would you have felt if you had had to register the birth and death of your baby?
How did you feel about registering the birth and death of your baby?
I wonder if you could tell me about whether there was any involvement from the coroner in understanding the death of your baby? [post mortem]
Were there any things that you would have found helpful that you didn't receive?
Care after birth
Can you tell me about any support you were offered by a midwife after the birth of your baby to allow you to talk about what you'd experienced?
What kind of discussions did you have with a doctor (obstetrician/neonatologist) after the birth?
Can you tell me about whether your GP visited you after you went home?
Understanding the death
Can you remember whether you were offered any investigations after your baby died to help understand their death? Who offered those to you?

What sort of information were you given about the possibility of undertaking investigations into why your baby may have died?
Could you tell me about any investigations you were aware of to understand why your baby died, like a post mortem?
How would you have liked the midwives and doctors to have helped you make the decision?
(try to find out whether neonatologists or obstetricians)
What did you find helpful in what they told you?
How did you feel about the decisions you'd made before the birth?

Returning to work

Before you were pregnant, can you tell me about whether you were working?
What sort of entitlements did you have to any maternity leave or sick leave after the birth of your baby?
How long was it before you felt prepared to return to work?
How did you feel about having to return to work at that stage?
How did maternity/ paternity leave and pay help you?
How would maternity/paternity leave have helped you do you think?
Have you been able to think yet about getting pregnant again? How did health professionals discuss with you about future plans of becoming pregnant

Final thoughts

Do you think of your loss as a miscarriage or a death? Some people feel that if they thought of their experience as a miscarriage it was easier to move on while other people said they felt very differently, that as a miscarriage they weren't recognised as being a parent or having a baby. How did you or do you feel?
Who have you sought support from in terms of friends, family, or support groups.

Messages for others

Are there any things you'd like to change for someone in the same position as you?
Are there any messages you'd like to give to the doctors and midwives that cared for you and your baby?
I'd just now like to give you the opportunity to tell me anything else you feel you'd like to add?
Just reflect for a moment and make sure you've told me everything you wanted to.
