# Supplementary material for: Parents’ experiences of care following the loss of a baby at the margins between miscarriage, stillbirth and neonatal death: a UK qualitative study
Source: BJOG. 2020 Feb 21;127(7):868–74. doi: 10.1111/1471-0528.16113 (PMC7383869; doi:10.1111/1471-0528.16113)
Supplement: Supplementary file 2 — Appendix S2 . Biographical profiles of parents participating in the study. [file BJO-127-868-s002.pdf]

Appendix S2. Biographical profiles of parents participating in the study

**Nesta:** During a scan at 23 weeks her baby's heartbeat could not be seen. Nesta's labour was induced and she gave birth to her son who showed no signs of life.
**Lisa and Matt:** Lisa had bulging membranes at 19 weeks and had stitches in her cervix to try to delay their baby's birth. At 22 weeks her waters broke and their baby was born alive and lived for two hours.
**Kareena and Raj**: Kareena experienced heavy bleeding from 16 weeks and went into labour at 21 weeks of pregnancy. Their son was born alive but died shortly after birth.
**Asun and David**: Asun had been feeling unwell for a few days, and her waters broke at 23 weeks. Their baby was born alive at 23+6 weeks and lived for three hours.
**Kamie and Dale**: Kamie went into preterm labour and her baby was born at 20 weeks showing no signs of life. Two years later, Kamie and Dale's daughter was born alive at 21 weeks but died shortly after birth.
**Kirsty and Matthew:** Kirsty was rushed to hospital after she went into labour at home at 23 weeks of pregnancy. Their daughter was born showing no signs of life.
**Elaine and David**: Elaine could no longer feel her baby moving and a scan showed the baby had died at 23 weeks. Elaine and David's baby was born showing no signs of life at 23 weeks and 6 days.
**Maxine and Steve**: Maxine's waters broke at 18 weeks and so her labour was induced early. Their baby was born showing no signs of life at 21 weeks of pregnancy.
**Joelle and Adam**: Joelle went into premature labour at 21 weeks. Their son was born showing no signs of life.
**Michelle and Ian:** Michelle started bleeding at 20 weeks of pregnancy and a scan showed her baby had died. Their baby was born showing no signs of life at 20 weeks.
**Emily and Mike**: Emily had heavy bleeding from 7 weeks of pregnancy. At 20 weeks Emily's waters broke and she went into premature labour. Their baby was born showing no signs of life at 20 weeks.
**Liz**: After heavy bleeding Liz went into labour at 20 weeks. Her twin babies were born showing no signs of life.
**Collette**: Collette's son was born at 20 weeks of pregnancy. She saw her son's heart beating but he wasn't breathing when he was born and was not registered as a live birth.
**Helen Z**: Helen had a scan at 23 weeks and her baby's heartbeat could not be seen. Her labour was induced and her baby was born showing no signs of life at 23 weeks.
**Sarah**: Sarah had a scan at 22 weeks and her baby's heartbeat could not be seen. Sarah's labour was induced and she gave birth to her son who showed no signs of life.
**Kerry**: Kerry had a scan at 23 weeks and her baby's heartbeat could not be seen. Kerry's labour was induced and she gave birth to her baby who showed no signs of life at 23 weeks.
**Sam**: At a scan at 20 weeks Sam found out her baby had a severe congenital anomaly. Her labour was induced and she gave birth knowing her son would not survive. He was born showing no signs of life.
**Camille**: Camille had frequent pains in her uterus from 18 weeks of pregnancy. Her waters broke and she went into labour at 21 weeks. Her daughter was born alive but died 53 minutes after birth.
**Loretta**: At a 20 week scan Loretta found out her baby had no heartbeat. Loretta's labour was induced and she gave birth to her son who showed no signs of life.
**Carly**: Carly had a scan at 23 weeks and her baby's heartbeat could not be seen. Carly's labour was induced and she gave birth to her daughter who showed no signs of life at 23 weeks.
**Alison**: Alison's 21 week scan showed her baby had no kidneys. Her labour was induced early and she gave birth to her son early knowing he would not survive. He was born showing no signs of life.
**Kelly**: Following bleeding at 23 weeks, a scan showed Kelly's baby did not have a heart beat. Her labour was induced and her baby was born showing no signs of life.
**Vikki Z**: At her 20 week scan Vikki found out her baby did not have a heartbeat and she gave birth to her two days later. Her baby was born showing no signs of life.
**Courtney**: At 20 weeks Courtney went into preterm labour. Courtney felt she saw her baby breathing but he was not registered as a live birth.

**Vikki**: Vikki discovered at her 20 week scan that her baby had no heartbeat and had died. She gave birth a few days later and her baby showed no signs of life at birth.
**Helen:** At Helen's 20 week scan severe health problems were identified with her baby. Her baby died following a high-risk procedure to try to help her. Her baby was born showing no signs of life at 23 weeks.
**Sharon**: Sharon experienced the loss of three sons between 19 and 22 weeks of pregnancy. In all three of her pregnancies she went into premature labour and her sons were all born showing no signs of life.
**Lindsay**: Lindsay's pregnancy was induced when she had a life-threatening infection that led to preterm labour. Her baby was born with a heartbeat but did not take a breath and was not registered as a live birth.
