# Supplementary material for: Parents’ experiences of care following the loss of a baby at the margins between miscarriage, stillbirth and neonatal death: a UK qualitative study
Source: BJOG. 2020 Feb 21;127(7):868–74. doi: 10.1111/1471-0528.16113 (PMC7383869; doi:10.1111/1471-0528.16113)
Supplement: Supplementary file 3 — Appendix S3 . Parents’ narratives of healthcare experiences. [file BJO-127-868-s003.pdf]

Appendix S3. Parents' narratives of healthcare experiences

**The importance of terminology**
"And just being told that they're not really alive because they're not going to survive, doesn't make it any easier. My advice would be - stop using the word 'miscarriage' with parents. Use the word 'having a baby', 'losing a baby'. Rather than 'you're having a miscarriage'. Because it doesn't prepare you for it. … So I think it's more about the language that people use, that they should be very, very careful about. It's one thing to have it written down in the notes, because that's how you communicate with other health professionals. But when you communicate to the parents, you need to show compassion." Camille<br><br>*"I can't compare what an earlier loss would feel like. I imagine it feels pretty awful. So I'm not trying to demean people who go through miscarriages, or make their grief insignificant. I just think the grief might feel the same, but the experience is so different, and the trauma is a lot different."* Carly<br><br>*"I remember the nurse saying that she looked great when she was born. And she was, she was - she was exactly what you'd expect a baby to look like, but just smaller, like everything was there in proportion… Like I mean, it wasn't like a - like a fetus, you know, they're slightly still developing. Like she, she was ready. All she was going to do from there was get bigger. She was perfectly formed. So…fingernails, and little things like that, as well. Just these tiny details that - to me, that's - that wasn't a miscarriage. In no way, shape or form. Medically it was... I think that terminology - I don't know how you'd, on earth you'd go around changing it. But I do feel like it doesn't do justice to what we went through."* Mike
**Preparedness for birth**
"The fact that I had to go through the whole process of giving birth and everything… I didn't realise how big he was going to be, and how formed he was... - Because they kept saying to me, 'Oh, it's a miscarriage. It's a miscarriage.' Because obviously before 24 weeks, they don't count it as a live baby. But so in my head I was like: 'it's not going to be like this, it's going to be like, like just blood or whatever'. But you know, I had to full on give birth, and they had to take the baby out. And then I had to give birth to the placenta." Courtney<br><br>*"I remember feeling really confused, and going, 'Well, why am I going to deliver the baby?' I just didn't understand at the time. It didn't compute that actually I was having a baby. I think in the back of my mind I thought that they'd be able to put her back in, or... Because she wasn't ready to come out. We got to the delivery suite. And they had a crash team ready for her. And the room was just full of people. And they were all desperately trying to get her out. They were pushing my stomach. They were telling me to push. And I, and I also remember they kept saying to me, 'Bear down. Bear down.' I didn't know what that meant. I hadn't been to any antenatal classes yet because we hadn't got that far along."* Kirsty<br><br>*"I still had to push and everything… Because I said to the bereavement midwife afterwards, I said something like, 'Gosh, that was awfully hard work - I can't imagine what it's like to really give birth.' And she said, 'But you did.' She said, 'You still had to get to...' I think I had to get to six centimetres dilated or something. And she was like, 'That's the majority of the hard work, it's just the same, and you did really well.'… And again, because she was small, you sort of think you don't, but I still had the awful contractions, I had to do the breathing, and still had to push, and all the - you know - I had to deliver the placenta afterwards. I still had to do exactly the same, it's just that she was small. But I don't think I was ready for that."* Emily<br><br>*"So they admitted me, this time to the maternity unit, which I was very pleased about. Because I was 20 weeks, I was like, 'Yes! Not going back to gynaecology.' And there, I was obviously looked after by midwives. I was treated really kindly. I was treated like a pregnant woman with a baby.*

Whereas in the early pregnancy unit, I was treated like a woman with a pile of tissue in her uterus. And whether that tissue was alive or not, they really didn't care. Whereas in the maternity unit, despite the fact that she still wasn't viable, they treated me completely differently. And they really cared. And there was compassion. And all those things that I never got when I was in the early pregnancy unit." Camille
**Preparedness for seeing the baby**
"We'd decided we didn't want to see him straight away. We wanted to see photos. Because we didn't know what to expect. And it's easier to forget a photo than it is something you've actually lived. So we said… 'We don't want to see him. But we would really like to see some photos.' And so our midwife, bless her, she - even though she'd finished work, she took some photos… and she showed us the photos. And she was like, 'He's, he's beautiful.' And so we looked at the photos, and then were like, 'Yeah. We would like to see him.' And so they brought him in, in a little - in a little basket, with a blanket over him… And so I think we saw, saw him five or six times throughout the next few days." Sam<br><br>*"And we were given options - you know - what we wanted to do. And we decided that we didn't want to see him straight away… I was really worried about what he would look like, because we knew at that point that he'd been dead for about a week. So I was really scared … of what he looked like. So we decided…when I gave birth - we won't look at him. We just kept our eyes closed the whole time. …And we asked for the midwife to make a decision about whether we would see him afterwards or not. And whether she thought that it was wise for us to see him afterwards. And she said 'I think,' she went 'No, I think - you know, he's absolutely fine. You should, you know, you could see him.' So then my husband and I - he - I asked him to go first. So he went first. And then they brought him out."* Sarah<br><br>*"My husband was actually really surprised when they put her in my arms, and he said, 'She's a real baby. She's even got hair.'… I don't think either of us were expecting to have a baby. Because we had been told the word 'miscarriage' so many times. Yeah. It wasn't. It really didn't register in our mind that we were having a baby. And that really, that moment really made me realise that when my husband said that - because I thought 'Yes, we're having a baby, and they should have prepared us for that, they really should have'. And instead of using that horrible word 'miscarriage', they should have said you're about to have a baby. And that probably would have helped a little bit."* Camille
**Memory-making**
"It's just like insult to injury, isn't it? Same with the birth certificate. We got a little, what was classed as a 'certificate of life'. But really, it's just a printed out bit of paper that the hospital gives you, that's not formal. It's not recognised, and it's not official, like a birth and death certificate. I think they just give your baby that bit of, bit more dignity... Rather than - you wouldn't feel that you'd need to explain it so much, or like how you're feeling. It's like a...and it's almost like a recognition of your grief. Like if you've got that birth and death certificate, like they were here and then they died. And like the way you're feeling is valid. Whereas it doesn't feel as valid if you don't have those." Carly<br><br>*"We got given like a fake birth certificate. And I know that some people would probably love that. And I know that that was given to us because they wanted us to know that they recognised we'd just had a baby. I don't want a fake birth certificate. And I think out of the whole experience, my hang-up has been the birth certificate. She was two days short. They had a crash team there. We had a baby, but she'll never be recognised by UK law. She'll never - she just didn't exist. And people treat you differently. People do treat it like you've had a miscarriage. And they'll tell you about their experiences of having a miscarriage. And I'm not going to take anybody's pain away, and say that my pain was worse than anybody else's. Because it's a very individual thing. But it's just a*

different experience. And I don't think you can put them in the same box. And having a, a loss of a baby at fewer weeks and having one where you have got a baby, they are different experiences. Same kind of pain, but different experiences. But I was all of a sudden in this other box that I didn't want to be in.” Kirsty

The one thing that's like really important to me, which I keep, in her memory box - like I keep all her stuff in, but the most important thing in there is her little wristband. They put the wristband on her. You know, saying 'baby Josephine'. You know? 'Mother Carly' on her little band. That's like the only real medical documented proof that I have that she was here. And so that band's so precious now, because I don't have anything else. Except her - I've got like a little document, like I got with her ashes. But those are the only official things that I have of her.” Carly

“And they encourage you, ‘Do you want me to take pictures? You will, you will appreciate these pictures. Not now, not tomorrow, but in, in, in the future you will appreciate that.’ And they're right. They're right. Because I look at the pictures few times at home. And, and I'm glad that they took the pictures of him. And, and also they done handprints and footprints. And yeah, few things that we - we actually can - somehow can - You can feel him, no? You can see him…” David

“I saw her a couple of times. I spent all day - all, all day with her on the, like the first day. And then a couple of hours the day after... But then after that, I said, ‘Look, I don't want to see the baby anymore.’ Because in that 24 hours, I could see like the baby's appearance had deteriorated slightly. And I thought if I see the baby any more after that, it's going to - the baby's going to look worse, and I wanted to remember her how she was. She was perfectly formed. But she was probably about the size of my hand.” Kelly

“In hindsight I'm actually disappointed that we didn't do more, because we wasn't told that we could… So we got to see him for about an hour. They took him. And then we signed the piece of paper, signing him over. And that was just like the last time that we saw him. Whereas now when I talk to people, stuff was said to them a lot clearer. Like stuff that they could do. Like they had pictures taken, and they had prints made of their hands and feet. And just little things. And I just feel really hard done by, because this one hospital could have done so much.” Courtney

Postnatal experiences

“Physically you walk out of the hospital, and you bleed and you clot like you would as a new mum. You, your milk comes in like a new mum. But you don't have a baby. So you've got all - and obviously your hormones are all over the place. And you have all that to go along with. And I think… as a new mum the first time round, you don't notice all that happening, because you've got a baby to focus on. But when you've got time, you notice everything... your body is so, so cruel. Because my body thinks it's just had a baby. And it doesn't have a baby.” Maxine

Longer-term emotional experiences

“it was nice that they were treating him as a baby, a child that we had lost, rather than a miscarriage and I think these are all the kind of things that made a massive difference to how we dealt with it afterwards… I found afterwards, there's a massive difference between my experience and a lot of other people, other people I've spoken to when I've been to - we went to a bereavement, a bereavement group. And I knew a lot of other people who'd had babies similar time as us, similar week, but had been treated - you know - not badly, but they felt that they had been treated as if they'd had a miscarriage. Whereas actually I felt - we very much felt like they had treated us as if we'd had a stillborn baby. Even though that wasn't the medical, you know, term.” Sarah

“I think it's almost like because she hadn't reached 24 weeks, it wasn't legitimate, it was - because it was still termed as a miscarriage. And because there hadn't been a birth certificate or a death certificate… it's almost like it was minimised and wasn't that big of a deal. Not just for the people

who were involved at the hospital, but for like families as well. They didn't really seem to grasp what had happened, or how horrific it had been. Or that you know, a few days here and there shouldn't really make much difference. I mean, I think if she'd have been born at 24 weeks, I think Kirsty would have been able to have like some maternity leave which I'm sure would have really helped her. She'd have very much appreciated that extra time off work. As it was, she was - she was back there herself after a couple of weeks. So I don't know, I'm sure it wouldn't have helped the loss or the grief to have had the paperwork, but I think perhaps how other people dealt with it, they might have been a bit more sympathetic, I guess is the word. A bit more understanding, that we'd been through something quite, quite bad." Matthew
